# Identification of VIMP as a gene inhibiting cytokine production in human CD4+ effector T cells

**DOI:** 10.1016/j.isci.2021.102289

**Published:** 2021-03-09

**Authors:** Christophe M. Capelle, Ni Zeng, Egle Danileviciute, Sabrina Freitas Rodrigues, Markus Ollert, Rudi Balling, Feng Q. HeFeng

**Affiliations:** 1 Department of Infection and Immunity, Luxembourg Institute of Health (LIH), 29, rue Henri Koch, 4354 Esch-sur-Alzette, Luxembourg; 2 Faculty of Science, Technology and Medicine, University of Luxembourg, 2, avenue de Université, 4365 Esch-sur-Alzette, Luxembourg; 3 Luxembourg Centre for Systems Biomedicine (LCSB), University of Luxembourg, 6, avenue du Swing, 4367 Belvaux, Luxembourg; 4 Department of Dermatology and Allergy Center, Odense Research Center for Anaphylaxis (ORCA), University of Southern Denmark, Odense, 5000 C, Denmark; 5 Institute of Medical Microbiology, University Hospital Essen, University of Duisburg-Essen, 45122 Essen, Germany

**Keywords:** Immunology, Cell Biology, Systems Biology

## Abstract

Many players regulating the CD4^+^ T cell-mediated inflammatory response have already been identified. However, the critical nodes that constitute the regulatory and signaling networks underlying CD4 T cell responses are still missing. Using a correlation-network-guided approach, here we identified *VIMP* (VCP-interacting membrane protein), one of the 25 genes encoding selenoproteins in humans, as a gene regulating the effector functions of human CD4 T cells, especially production of several cytokines including IL2 and CSF2. We identified VIMP as an endogenous inhibitor of cytokine production in CD4 effector T cells via both the E2F5 transcription regulatory pathway and the Ca^2+^/NFATC2 signaling pathway. Our work not only indicates that VIMP might be a promising therapeutic target for various inflammation-associated diseases but also shows that our network-guided approach can significantly aid in predicting new functions of the genes of interest.

## Highlights

• VIMP is temporally upregulated after TCR stimulation in human CD4 effector T cells
• VIMP inhibits cytokine expression in human CD4 effector T cells
• VIMP inhibits cytokine expression via the NFATC2/Ca^2+^ signaling pathway
• VIMP inhibits cytokine expression by controlling E2F5 expression

## Graphical abstract

**Figure:**
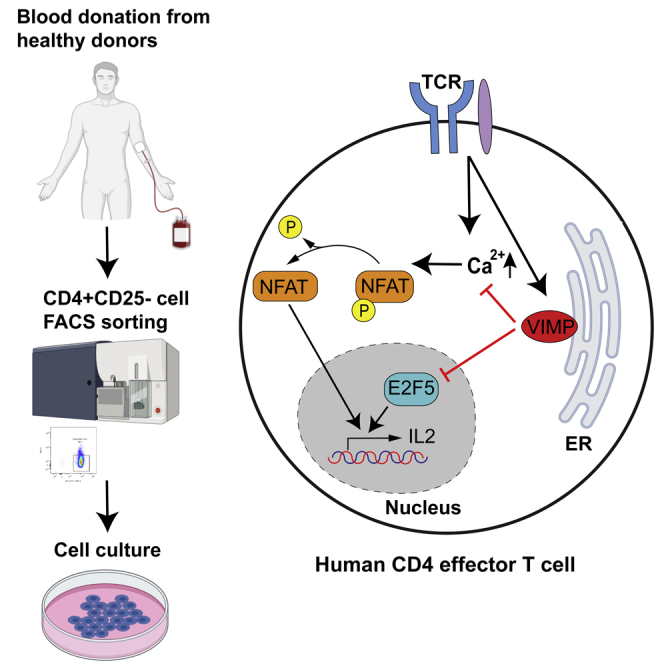

## Introduction

CD4^+^ T cells represent a major subset of immune cells that are crucial for mounting and regulating an adequate immune response. However, during many infectious and complex chronic diseases, those T cells are dysregulated, either having an impaired responsive capacity or causing adverse effects through self-recognition and/or overactivation. Therefore, rebalancing the CD4^+^ T cell-mediated inflammatory response has been essential for the design of therapeutic options for those diseases (Zhu and Paul, 2010a). Although many players regulating the inflammatory response, cytokine production, and differentiation of CD4^+^ T cells have already been identified in the past (Brownlie and Zamoyska, 2013; Rodriguez-Jorge et al., 2019; Saez-Rodriguez et al., 2007; Zhu and Paul, 2010b), a thorough understanding of the regulatory and signaling networks governing inflammatory cytokine production in T cells is still missing. The gap is not only attributable to the long-standing nature of traditional trial-and-error experimental procedures but also to the lack of reliable high-throughput computational prediction.

VIMP, also known as NCBI: Selenoprotein S (SELS), SELENOS, TANIS, or SEPS1, is one of the only 25 genes encoding the 21st amino acid selenocysteine in humans (Schomburg, 2011). Located in the endoplasmic reticulum (ER) membrane, VIMP is mainly known as an important component of the ER-associated degradation (ERAD) complex (Kim et al., 2007; Qin et al., 2016) and physically binds to several ER membrane proteins (Lee et al., 2015; Ye et al., 2005). VIMP plays a role in mediating retro-translocation of misfolded proteins from the ER lumen to the cytosol, where the ubiquitin-dependent proteasomal degradation takes place (Ye et al., 2004). Genome-wide association studies have shown that polymorphisms in the promoter region of *VIMP* are linked to a wide spectrum of common complex diseases, including cardiovascular disease (Alanne et al., 2007), diabetes (Karlsson et al., 2004; Olsson et al., 2011), cancer (Meplan et al., 2010; Shibata et al., 2009; Sutherland et al., 2010), sepsis (He et al., 2014), and autoimmune diseases (Santos et al., 2014; Seiderer et al., 2007), in which activation of the immune system is believed to be dysregulated (Kuchroo et al., 2012).

Meanwhile, dysfunction of the ER and the unfolded protein response causes intestinal inflammatory diseases in several murine models (McGuckin et al., 2010). Additionally, a reduced expression of VIMP causes an increased expression of inflammatory cytokines, such as NCBI: IL6, IL1β, and TNFα in macrophages (Curran et al., 2005), as well as IL1β and IL6 expression in astrocytes (Fradejas et al., 2011). However, other studies did not show significant association between *VIMP* and the examined human inflammatory diseases (Martinez et al., 2008). This controversy underlines the necessity for a better understanding of how *VIMP* contributes to the pathogenesis of some inflammatory diseases, i.e., through which cell types and which molecular pathways *VIMP* contributes to the observed dysregulated inflammatory responses. Therefore, we sought to investigate whether and how *VIMP* plays a role in relevant specific immune cells, e.g., CD4^+^ T cells, a key subset of immune cells orchestrating different types of immune responses and being heavily involved in different complex diseases, as well as infectious diseases, such as COVID-19 (Braun et al., 2020; Mathew et al., 2020).

We have previously developed a correlation-network-guided approach, based on the guilt-by-association theory (Beyer et al., 2007; Gillis and Pavlidis, 2011; Oliver, 2000), to predict novel key genes of a given biological process or function and have successfully applied it to human CD4^+^CD25^high^CD127^low^ regulatory T cells (Tregs) (Danileviciute et al., 2019; He et al., 2012). Here, we extended the strategy to human CD4^+^ effector T cells (Teffs) that were derived and expanded from sorted CD4^+^CD25^-^ T cells by co-culturing with EBV-transformed B cells and were able to predict that VIMP might play an important role in regulating the effector responses of Teffs. Combining both the network analysis and experimental verification, we identify VIMP as a previously unreported vital endogenous inhibitor of cytokine production in human CD4^+^ Teffs and reveal the molecular mechanisms through which VIMP regulates CD4^+^ Teff responses.

## Results

### VIMP is temporally upregulated following TCR stimulation in Teffs

**Figure 1:**
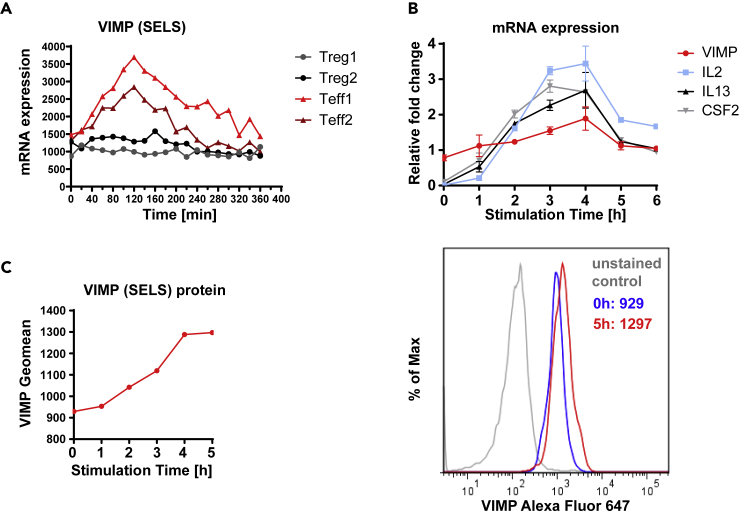
VIMP is temporally upregulated in Teffs following TCR stimulation (A) The kinetics of transcriptional expression of VIMP in the first 6 h following anti-CD3/-CD28 stimulation assessed by HTR time-series microarray data. Teff1 and Teff2 are the two independent repeated HTR time-series experiments from different donors. (B) Representative experiments, reproduced in 4 independent donors showing mRNA expression of *VIMP*, *IL2*, *IL13,* and *CSF2* measured by qPCR in Teffs stimulated by anti-CD3/-CD28 beads. The data represent the gene expression normalized to *RPS9*. Data are mean ± standard deviation (SD). (C) Representative flow cytometry quantitative analysis showing elevation of VIMP protein expression in Teffs following TCR stimulation. Results represent four (B) and three (C) independent experiments of different donors.

Using our previously reported high-time-resolution (HTR) time-series transcriptome data of Tregs and Teffs following TCR (T cell receptor) stimulation in the first 6 h (He et al., 2012), we observed that the transcript level of VIMP in Teffs temporally peaked within 2–3 h following stimulation, which was followed by a gradual decrease (Figure 1A). In contrast, the *VIMP* mRNA level was kept almost constant in Tregs during the first 6 h following TCR stimulation (Figure 1A), indicating a possible specific role for VIMP in Teffs. Our quantitative real-time PCR (qPCR) results validated the transitionally elevated expression of the VIMP transcript in Teffs isolated from different healthy donors (Figure 1B). We also observed a correlation over time between the transcription levels of NCBI: *VIMP*, *IL2*, *IL13,* and *CSF2 (*GM-CSF) following TCR stimulation, indicating a potential regulatory relationship between *VIMP* and some of the cytokines in Teffs (Figure 1B). By flow cytometry (Figure 1C), we confirmed the gradual upregulation of VIMP protein expression in the first 5 h following TCR stimulation. In summary, both mRNA and protein expression of VIMP were upregulated following TCR stimulation, which was correlated with the expression of several examined cytokines, indicating a potential role of VIMP in regulating Teff responses.

### VIMP inhibition upregulates cytokine expression in Teffs

**Figure 2:**
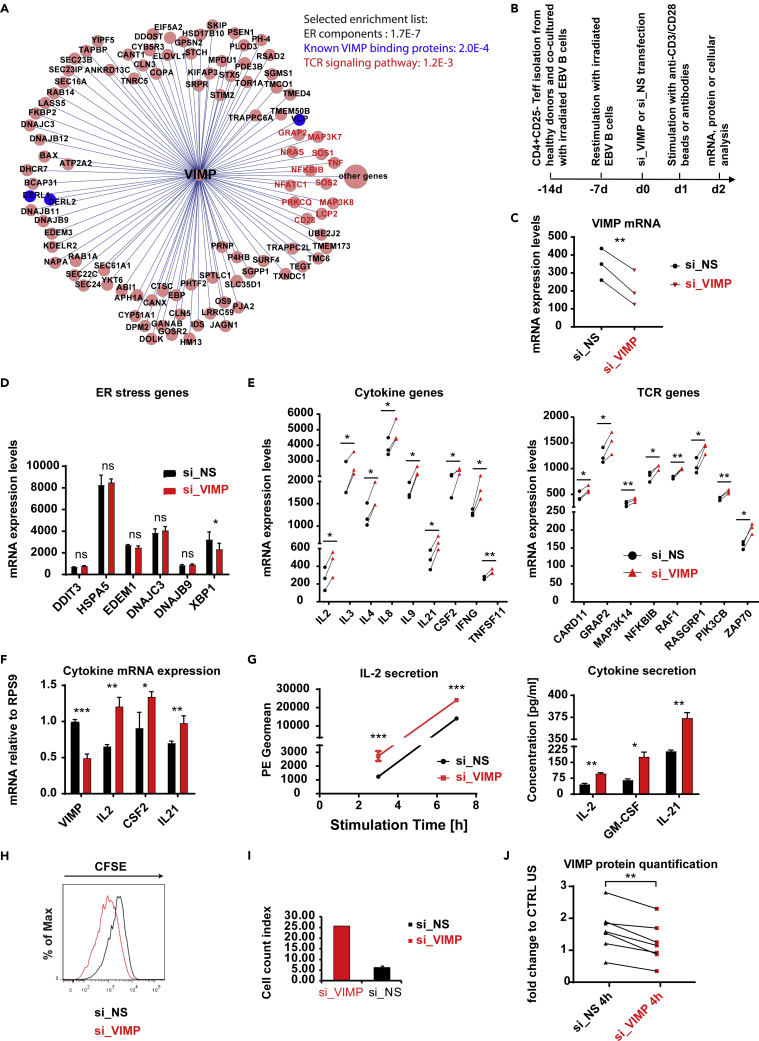
VIMP controls cytokine expression in Teffs and interferes with the TCR signaling pathway (A) The *VIMP* subnetwork extracted from the constructed Teff correlation network based on the HTR transcription microarray data. Each circle represents one gene. Each line between *VIMP* and the other genes represents a correlation link. The selected list of significantly enriched pathways or components is displayed (the p value resulted by cumulative binomial distribution test was provided for each item). (B) Schematic of the experimental flow for the stimulation, gene silencing, and analysis of Teffs. (C) mRNA expression showing the significant knockdown of VIMP in the microarray experiment. (D and E) Microarray data showing the fold change or expression values in the mRNA of ER-stress responsive genes (D) and cytokine and TCR signaling genes (E). We only presented the transcripts with p values lower than 0.05 by both PLIER and RMA methods and at least a 1.2-fold change in all 3 independent donors (for details, see methods). (F) mRNA expression measured by qPCR of the genes *VIMP*, *IL2*, *CSF2,* and *IL21* of Teffs following TCR stimulation and knockdown with either control non-specific scrambled siRNA (si_NS) or *VIMP*-specific siRNA (si_VIMP). Before stimulation, the cells were first transfected with siRNA for 1 day (for all the figures). (G) The concentration of the cytokines IL2, GM-CSF, and IL21 detected in the cell culture medium following anti-CD3/-CD28 stimulation for different time points (left panel, IL2 alone by CBA measurement) or 8 h (right panel, multiplexing by MSD). PE geometric mean (Geomean) corresponds to the IL2 concentration signal in the media. (H and I) Proliferation of Teffs following VIMP knockdown and TCR stimulation, measured by CFSE proliferation assay (H) and by counting the T cells following stimulation (I). Before Teffs were co-cultured with EBV-transformed B cells for 2 days, they were first transfected with siRNA for 1 day. (J) Quantification of the western blot protein bands and normalization of VIMP to the housekeeping gene GAPDH. Each dot represents one sample. CTRL US, unstimulated Teffs treated with si_NS. Data are mean ± SD. The p values are determined by a two-tailed paired Student's t test (except for A and G). The results in (G) were analyzed using non-paired t test. ns or unlabeled, non-significant; ∗p<=0.05, ∗∗p<=0.01, and ∗∗∗p<=0.001. Results represent three (C–E) and six (F–J) independent experiments of different donors. See also Figure S1.

The upregulation of VIMP and its correlation to cytokine expression encouraged us to further investigate VIMP's potential role in CD4 T cell responses. We and others have previously shown that the enriched pathways, processes or functions among the genes surrounding a given hub gene in the correlation network might give valuable indications on potential new functions of the given hub gene (Danileviciute et al., 2019; He et al., 2012). Therefore, we used our correlation network-guided approach to predict the potential functions of *VIMP* by identifying the enriched pathways among the genes that are linked to *VIMP* within the subnetwork of the Teff correlation network, which was extracted from our published HTR datasets and networks (He et al., 2012) (Figure 2A).

Consistent with its known function and its localization in the ER membrane, the genes surrounding *VIMP* in the correlation network were significantly enriched for ER components (p value *=* 1.7 × 10^−7^, cumulative binomial distribution) (Figure 2A). Furthermore, 3 of the 10 experimentally validated VIMP-binding partners found in the literature in other cellular types are directly linked to *VIMP* in the Teff correlation network (p value = 2.0 × 10^−4^, http://string-db.org [Szklarczyk et al., 2019]), indicating the reliability of our method. Surprisingly, the pathway enrichment analysis shows that the genes linked to *VIMP* are significantly enriched for components involved in the TCR signaling pathway (p value *=* 1.2 × 10^−3^, cumulative binomial distribution) (Figure 2A), suggesting a potential role of VIMP in the Teff response according to our network-based analysis strategy (Danileviciute et al., 2019; He et al., 2012). However, the genes linked to the hub gene of interests in the correlation network could follow at least two scenarios (He and Ollert, 2016; Langfelder and Horvath, 2008; van Dam et al., 2018). First, those genes could be co-regulated by chance with the hub gene and perform independent functions. Second, those genes could be co-expressed with the hub gene and play related roles in the same pathways to coordinate cellular resources for a particular function or purpose under certain conditions. We will test these possibilities in this work.

To systematically assess whether and how *VIMP* controls the inflammatory response of Teffs, we performed a transcriptome analysis of CD4 Teffs isolated from peripheral blood mononuclear cells (PBMCs) of three healthy donors that were subjected to a specific-small interfering RNA (siRNA) knockdown of VIMP (si_VIMP) or a control unspecific scrambled siRNA (si_NS) followed by anti-CD3/-CD28 stimulation (Figure 2B). As shown in Figure 2C, the mRNA expression of *VIMP* was significantly downregulated in Teffs by using siRNA knockdown.

As VIMP has reported functions in ER stress, we first checked the ER-stress responsive genes in the transcriptomic data of the Teffs transfected with si_VIMP versus that treated with control siRNA (si_NS). By perturbing the expression of *VIMP*, we expected a change in the expression of some ER-stress responsive genes. Nonetheless, our transcriptome data of Teffs with VIMP partial knockdown did not show any significant change in mRNA expression of those genes (e.g., NCBI: *CHOP [DDIT3]*, *GRP78 [HSPA5]*, *EDEM1*, *DNAJC3 [P58IPK],* and *DNAJB9 [ERdj4]* [Lee et al., 2003]) (Figure 2D). Only the expression of the ER-stress regulator *XBP1* (Yoshida et al., 2001) was significantly but modestly decreased. Indeed, data from intestinal epithelial cells show that VIMP is only a marker, but not a regulator, of ER stress (Speckmann et al., 2014). This shows that the direct involvement of VIMP in ER stress might not be ubiquitous to all cell types. We therefore ruled out the possibility that VIMP directly regulates the expression of the ER-stress responsive genes, indicating other roles of VIMP in modulating the Teff responses.

Considering that the TCR signaling pathways were significantly enriched in the VIMP correlation network, we further analyzed the genes related to the TCR signaling pathway in Teffs after *VIMP* knockdown. Notably, we found 13 significantly upregulated genes involved in the TCR signaling, including several cytokines, namely, NCBI: *IL2*, *IL4*, *CSF2,* and *IFNG* (refer to https://www.genome.jp/kegg-bin/show_pathway?hsa04660) in the microarray datasets of the Teffs, although only subjected to a partial knockdown of *VIMP* (Figure 2E). Moreover, transcripts of the key TCR-related signaling molecules, such as NCBI: *GRAP2*, *ZAP70*, *RASGRP1,* and *RAF1,* were significantly affected (Figure 2E). With the observation in mind that *VIMP* and the TCR signaling-related genes were directly linked in our HTR correlation network (Figure 2A), this effect of the siRNA perturbation was not unexpected. Our results suggest that VIMP negatively regulates the expression of specific cytokines and influences the expression of important components of the TCR signaling pathway.

To further confirm whether *VIMP* regulates cytokine expression in Teffs, using PBMC of independent donors we measured the cytokine mRNA expression by qPCR and the secreted cytokines of Teffs that were exposed to a *VIMP* knockdown. Indeed, NCBI: *IL2*, *IL21,* and *CSF2* mRNA were significantly upregulated in stimulated Teffs transfected with si_VIMP, compared with control Teffs (with si_NS) (Figure 2F). This observation was further consolidated by increased IL2, IL21, and GM-CSF protein levels in the culture media of stimulated Teffs transfected with si_VIMP, compared with that treated with control scrambled siRNA (Figure 2G). Furthermore, the VIMP knockdown also significantly promoted T cell proliferation as indicated by both carboxyfluorescein succinimidyl ester (CFSE) peak shifting and Teff cell number counting experiments (Figure 2H and 2I). As IL2 concentration was already significantly higher at 3 h following stimulation (Figure 2G) and no cell division was expected, the enhanced IL2 secretion following VIMP knockdown was not simply caused by more Teffs. All the analyses were done under the precondition that VIMP protein was successfully silenced (Figure 2J). In short, VIMP negatively regulates the expression of several cytokines in Teffs following stimulation.

Considering that *VIMP* encodes selenocysteine, thus requiring selenium (Se) for its protein synthesis, and the fact that a relatively low concentration of Se was used in our T cell media (IMDM, ∼0.066 μM), we next supplemented sodium selenite in the T-cell culture media to the range of physiological concentrations (∼1 μM) (Rauhamaa et al., 2008; Safaralizadeh et al., 2005; Stranges et al., 2011). In line with the reported inverse association between Se status and inflammatory bowel diseases (Kudva et al., 2015), increasing the concentration of Se in the media generated a dose-dependent suppressive effect on IL2 production of sorted CD4 Teffs following TCR stimulation (Figures S1A and S1B). Meanwhile, increasing Se concentration to a physiological concentration upregulated the VIMP expression of stimulated CD4 Teffs in three of five tested donors (Figures S1C and S1D). These results again indicate that Se, at least partially, negatively regulates the expression of cytokines, e.g., IL2 in CD4 Teffs via VIMP, which is in line with our knockdown results.

### VIMP controls cytokine expression via the transcription factor E2F5

**Figure 3:**
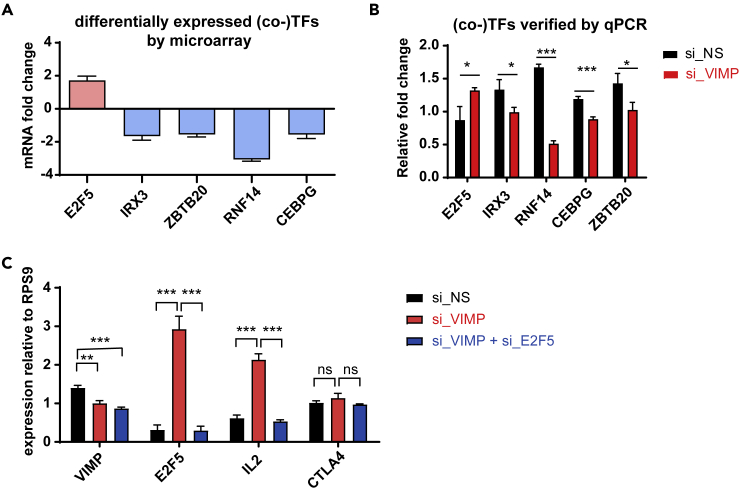
VIMP inhibits E2F5 to regulate IL2 expression (A) The most significantly affected (co-)transcription factors selected from our microarray analysis including three donors; the y axis indicates the fold change between Teffs transfected with siRNA specific against VIMP(si_VIMP) or non-specific scrambled si_RNA (si_NS). (B) mRNA expression measured by qPCR from Teffs of independent healthy donors of the genes displayed in (A) to confirm the change in their expression levels following *VIMP* knockdown. (C) mRNA expression measured by qPCR of the genes *VIMP*, *E2F5*, *IL2,* and *CTLA4* of Teffs transfected with si_NS, si_VIMP, or both si_VIMP and si_E2F5. Data are mean ± SD. The p values are determined by a two-tailed paired Student's t test. ns or unlabeled, non-significant; ∗p<=0.05, ∗∗p<=0.01, and ∗∗∗p<=0.001. Results represent four (B and C) independent experiments of different donors.

Next, we aimed to identify any (co-)transcription factors (TFs), whose expression were significantly affected after silencing VIMP, as they often serve as the key components orchestrating the activity of the relevant pathways. Through a systematic analysis of all the known mammalian TFs or co-factors (Zhang et al., 2012) in our microarray datasets, NCBI: *E2F5* was found to be the most significantly upregulated TF, following a partial VIMP knockdown (Figure 3A). Conversely, NCBI: *RNF14* (ring finger protein 14) was the most downregulated cofactor together with the downregulated TFs NCBI: *CEBPG* (CCAAT enhancer binding protein gamma), NCBI: *ZBTB20* (zinc finger and BTB domain containing 20), and NCBI: *IRX3* (Iroquois homeobox 3) (Figure 3A). We further confirmed the expression change of these (co-)TFs by qPCR in independent healthy donors (Figure 3B).

*E2F5* has been reported to be a downstream target of IL-2 in an immortalized human T cell line (Brennan et al., 1997). But to our knowledge, there are no reports yet of *E2F5* sitting at the upstream pathways regulating inflammatory responses, especially cytokine production. Nevertheless, being the most significantly upregulated TF following a partial knockdown of *VIMP*, we assumed that *E2F5* might be an important component in the regulatory pathway through which *VIMP* regulates the Teff inflammatory response.

Therefore, we decided to investigate whether *VIMP* controls the cytokine expression by negatively regulating *E2F5* expression in stimulated Teffs. To examine this hypothesis, we silenced *VIMP* alone or in combination with *E2F5* and measured the expression of selected cytokines by qPCR. In addition to the reduced expression of *VIMP*, the upregulation of *E2F5* expression that was driven by *VIMP* knockdown was abolished in the *VIMP* and *E2F5* double knockdown Teffs (Figure 3C). Silencing *VIMP* alone upregulated *IL2* expression in stimulated Teffs, whereas a dual knockdown of *VIMP* and *E2F5* suppressed the surge of *IL2* caused by VIMP knockdown alone (Figure 3C). Even though *E2F5* is a general regulator of transcription, we did not observe any effect of *E2F5* knockdown on genes that are not directly involved in Teff inflammatory response, such as NCBI: *CTLA4* (Figure 3C). This excluded a generalized effect of *E2F5* on the transcriptional regulation in Teffs. In brief, our data support the fact that *VIMP* regulates the expression of inflammatory cytokines, i.e., IL2, by restraining the expression of the TF *E2F5* in Teffs.

### VIMP controls cytokine expression via the Ca^2+^/NFATC2 signaling pathway

**Figure 4:**
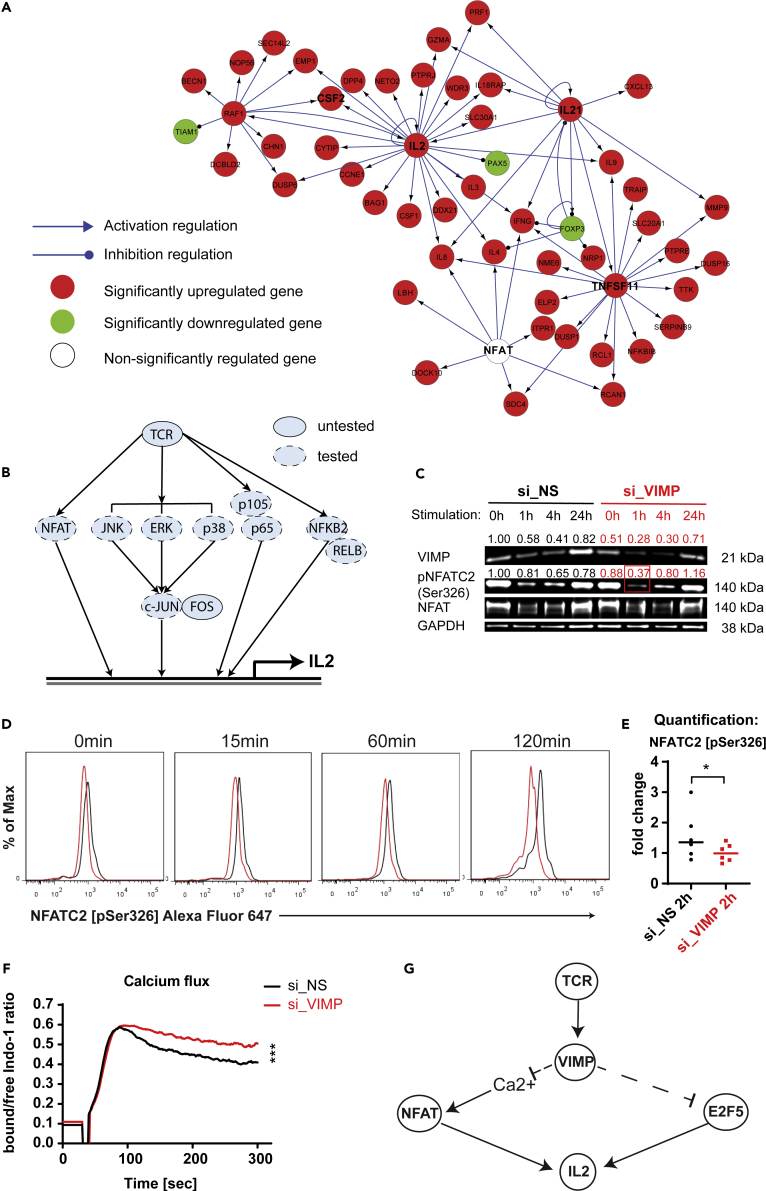
VIMP controls cytokine expression via the Ca^2+^/NFATC2 phosphorylation pathway (A) Network representation of the cytokine and TCR-related genes affected by the knockdown of VIMP by Ingenuity Pathway Analysis (IPA). Red, significantly upregulated genes; green, significantly downregulated genes; white, non-significantly affected gene at the transcriptional level. The link with arrow indicates a known direct or indirect positive transcription regulation; the link with circle indicates a negative one from the IPA knowledge databases. (B) Graphical representation of the major signaling pathways downstream of the TCR signaling, (un)tested for their phosphorylation levels. (C and D) Phosphorylation of NFATC2 (NFAT1) in Teffs assessed by western blot (C) or flow cytometry (D) at different time points following anti-CD3/-CD28 stimulation (C) or PMA/ionomycin stimulation (D). Before stimulation, Teffs were first transfected with specific siRNA against VIMP (si_VIMP) or non-specific siRNA (si_NS) for 24 h. (D) Representative flow cytometry plots of pNFATC2 in Teffs. (E) Pooled pNFATC2 data from multiple donors at 120 min post stimulation. For (D and E), only gated viable Teffs were displayed for all the phosphorylation results. The y axis represents the percentage of maximum (scales each curve to mode = 100%) (% Max). The fold change was calculated by normalizing the geometric mean (Geomean) of the fluorescence intensities of all the conditions to that of the unstimulated control siRNA knockdown condition. (F) Representative graph of 3 independent experiments for the calcium flux in Teffs following stimulation. The one displayed here used ionomycin stimulation after si_VIMP or si_NS transfection for 24 h. The y axis represents the ratio between calcium-bound and free Indo-1 dye over time. (G) Graphical representation summarizing the two mechanisms through which VIMP regulates cytokine expression in CD4 Teffs. Data are mean ± SD. The p values are determined by a two-tailed paired Student's t test. ns or unlabeled, non-significant; ∗p<=0.05, ∗∗p<=0.01, and ∗∗∗p <=0.001 . Results represent three (C and F) and six (D and E) independent experiments of different donors. See also Figures S2 and S3.

To further delineate VIMP's regulatory pathways beyond the altered expression of individual TFs determined by the differential expression analysis of our microarray datasets, we applied the Ingenuity Pathway Analysis (IPA) to map the up- or downregulated cytokine and TCR related genes into the known regulatory network structures. We found that many of those differentially expressed genes are controlled by the expression change of the so-called hub genes NCBI: *IL2*, *RAF1*, *IL21*, and *TNFSF11*, as well as nuclear factor of activated T cells (NFAT) activity (Figure 4A). Although *NFAT* transcript expression was not significantly affected (Figure 4A), its activity was predicted to be increased by the IPA computational analysis. Meanwhile, we investigated the *VIMP* subnetwork in the Teff correlation network in more depth (Figure 2A). We found that genes for several components of NF-κB, NFAT, and MAPK signaling pathways were also directly linked to *VIMP*, indicating that those pathways might be involved in the regulation of the inflammatory response of Teffs by VIMP. To determine whether any of the relevant signaling pathways downstream of the TCR pathway that were suggested by the computational analysis are affected by VIMP expression, we quantitatively assessed the phosphorylation levels of up to 10 various signaling proteins by flow cytometry (Figure 4B). Canonical (NFKB1, p105, and p65) and non-canonical (NFKB2, p100, and RELB) NF-κB signaling pathways, as well as several MAP kinase sub-pathways (ERK1/2, p38, JNK1/2, and cJun) were not significantly affected in their phosphorylation levels (Figures S2A–S2E and S3). The phosphorylation level in one of the NFAT family members, NFATC1, was also not significantly affected by VIMP knockdown in stimulated Teffs (Figures S2F and S3). However, the phosphorylation level at the specific site Ser326 of another NFAT family member, NCBI: NFATC2 (also known as NFAT1), was significantly reduced even following a partial VIMP knockdown, as quantified by both flow cytometry and western blotting in Teffs isolated from different donors (Figures 4C–4E and S2G). Total NFAT1 protein remained unaffected by the partial VIMP knockdown (Figure S2H). In resting T cells, NFAT proteins are phosphorylated and reside in the cytoplasm (Okamura et al., 2000; Sharma et al., 2011). To be able to translocate to the nucleus and induce gene expression, NFAT is de-phosphorylated following the TCR signaling. As the NFAT activity is known to regulate IL2 expression in T cells (Chow et al., 1999), the observed downregulation of NFATC2 phosphorylation, following *VIMP* knockdown, demonstrated that the upregulation of IL2 expression was, at least in part, due to an increase in NFAT activity.

The distinguishable feature of NFAT is that it relies on Ca^2+^ influx and subsequent Ca^2+^/calmodulin-dependent phosphatase calcineurin to become dephosphorylated and being able to translocate to the nucleus to induce gene expression (Hogan et al., 2003). Although *VIMP* has not yet been linked to the calcium signaling, other selenoproteins have been described to regulate the calcium signaling and homeostasis (Pitts and Hoffmann, 2018). We therefore further asked whether *VIMP* knockdown affects the calcium flux in Teffs and measured it by flow cytometry using the calcium indicator Indo-1. Indeed, the Teffs in which *VIMP* was silenced versus the control Teffs showed a significantly higher flux of Ca^2+^ ions (Figure 4F), further illustrating the increased NFATC2 activity and IL2 expression.

In summary, our data strongly suggest that *VIMP* inhibition upregulates the expression of cytokines, such as IL2, by two mechanisms at different levels (Figure 4G). On the transcription regulatory level, *VIMP* controls the expression of TF E2F5 and multiple genes involved in the TCR signaling and the inflammatory response. On the signaling transduction level, *VIMP* knockdown modulates Teff responses by controlling the Ca^2+^ flux and the downstream NFATC2 de-phosphorylation.

## Discussion

So far many important components in the regulatory or signaling networks modulating the inflammatory responses of Teffs still remain elusive. With the development of systems biomedicine, researchers have greater opportunities to use top-down approaches to objectively infer and identify novel key genes or proteins in the process of interest.

In this work, we have applied our previously published correlation network-guided strategy to predict new genes regulating the effector functions of CD4^+^CD25^-^ Teff cells, i.e., cytokine production. We identified VIMP, encoding an ER membrane-associated selenoprotein, as a previously unrecognized negative regulatory gene of the Teff response. VIMP is best known for its critical functions in ER stress, which was demonstrated in some tested cell types. Our transcriptomic correlation network in Teffs also indicates that VIMP might be involved in ER-stress-related functions. However, as shown here, inhibiting the VIMP expression in primary human Teffs did not support the fact that VIMP is critical for the transcriptional regulation of ER-stress responsive genes in Teffs. Next, the correlation network navigated us to check the TCR signaling pathways. As demonstrated in different layers, VIMP indeed substantially regulated the expression of several inflammatory cytokines, especially IL2, in Teffs. We next investigated the VIMP regulatory mechanisms using primary human Teffs isolated from different healthy donors, the most clinically relevant available materials. Combining the analysis of time-series correlation network with knockdown-based regulatory networks, we further predicted that VIMP might go through the NFAT signaling pathway, or MAP kinase or NFKB signaling pathways to mediate the effector functions of Teffs. After testing those signaling pathways one by one, we finally pinpointed that VIMP inhibition enhances cytokine production of Teffs via the NFATC2 signaling pathway. The involvement of the NFAT signaling pathway was further backed by the influence of VIMP inhibition on calcium (Ca^2+^ influx, which is vital to the activation of the NFAT signaling pathway). Coincidently, Joost and colleagues have recently reported the co-expression of *VIMP* and *NFATC2* transcripts within the murine interfollicular epidermis using single-cell RNA sequencing analysis, indicating from another angle that our conclusion might hold true (Joost et al., 2016). We have also shown that *E2F5* plays a significant role in the VIMP-mediated regulation of the Teff IL2 expression. However, whether the *E2F5* pathway and the Ca^2+^/NFATC2 signaling controls VIMP-mediated IL2 expression in a sequential manner or in parallel requires further investigation. Although the published association studies have already shown that the VIMP expression levels and/or SNPs are correlated with the risk of several types of diseases, it remains unsolved whether VIMP deficiency can regulate the effector functions of Teffs *in vivo*.

In our TF-focused analysis, we identified not only *E2F5* as the most upregulated TF but also several downregulated TF genes, following VIMP knockdown. Among those downregulated ones, *RNF14* (ring finger protein 14), a less characterized gene, represented the most significantly downregulated co-factor, attributable to VIMP knockdown in Teffs. Although very limited, a published report shows that *RNF14* modulates the expression of inflammatory and mitochondria-related genes in a murine myoblast cell line (Ingham et al., 2014). Another downregulated TF *ZBTB20,* originally studied in human dendritic cells (Zhang et al., 2001), and later in myeloid cells (Liu et al., 2013) and B cells (Zhu et al., 2018), has been shown to regulate their effector functions and differentiation. The Iroquois homeobox 3 (*IRX3)* has been recently linked to human CD8 T cell survival and fate determination *in vitro* (Persengiev, 2017). Although there is no direct evidence of *CEBPG* being involved in the regulation of cytokine expression in CD4 effector T cells, other C/EBP protein family members have been shown to act as negative regulators in the production of inflammatory cytokines (Berberich-Siebelt et al., 2000; Tanaka et al., 2014). Therefore, those TFs might deserve further investigation.

Selenoproteins fully rely on selenium for their biosynthesis and function. Dietary selenium supplementation in mice has been shown to increase the biosynthesis of several selenoproteins including SELS/VIMP (Stoedter et al., 2010; Tsuji et al., 2015) and to affect the expression of several inflammatory cytokines (Beck et al., 2001; Hao et al., 2016; Stoedter et al., 2010; Vunta et al., 2007; Zhu et al., 2017). Dietary selenium supplementation has further been linked to alleviate several complex and multifactorial diseases (Duffield-Lillico et al., 2003; Kim et al., 1999, 2000; Kudva et al., 2015). On the other hand, selenium deficiency might affect the synthesis of multiple selenoproteins in mice, resulting in an increased pathology from viral or bacterial infections (Beck et al., 2001; Gao et al., 2016). In our media (complete IMDM) for short-term T cell culture, the Se concentration (0.066 μM) was around 15 times lower than in human sera (∼1 μM) (Rauhamaa et al., 2008; Safaralizadeh et al., 2005; Stranges et al., 2011). Although the Se concentration used in our media was low, our western blotting results (Figure 4C) have demonstrated that the Se concentration was not yet a limiting factor for VIMP protein synthesis during the tested period of up to 24 h following stimulation, as the protein expression of VIMP still increased following TCR stimulation. In the VIMP-knockdown T cells, where the VIMP protein synthesis was further reduced, the low concentration of Se in the media was thus not a concern. Therefore, our conclusion derived from IMDM media with a VIMP-knockdown approach is reliable. Last but not least, increasing Se concentration showed a dose-dependent suppressive effect on IL2 production (Figures S1A and S1B). Following Se supplementation, the majority of the tested donors exhibited enhanced expression of VIMP in CD4 Teffs (Figure S1D). For the other donors, already having a high level of VIMP expression (Figure S1D), Se supplementation cannot further increase the expression of VIMP, but still inhibited cytokine production, possibly via enhancing the expression of the other selenoproteins as an alternative pathway. These observations indicate that at least for some patients with VIMP deficiency, Se supplementation would show beneficial values in suppressing pro-inflammatory cytokine responses of CD4 T cells.

Interestingly, the immune system presents a sexual dimorphism (Klein and Flanagan, 2016), where females appear to have a stronger humoral and cellular immune response in general, making them more resistant to infectious diseases (vom Steeg and Klein, 2016), nevertheless, more susceptible to autoimmune diseases (Angum et al., 2020; Jacobson et al., 1997). CD4 T cells, the focus of this study and the central orchestrators of immune responses, also show a differential sex-specific regulation (Afshan et al., 2012; Aldridge et al., 2018; Klein and Flanagan, 2016). Multiple factors on the genetic (Bianchi et al., 2012; Cacciari et al., 1981; Kocar et al., 2000), hormonal (Straub, 2007), or environmental level (Jensen et al., 2016; Kawai et al., 2010) have been shown to regulate sex-specific effects in immune responses. It is worth noting that selenium also displays intriguing sex-specific differences in regard to its metabolism (Seale et al., 2018), tissue distribution (Pitts et al., 2015), and effects in several physiological and pathological conditions, including immune-associated diseases (Li et al., 2020; Lu et al., 2019; Riese et al., 2006; Schomburg and Schweizer, 2009; Stoedter et al., 2010; Waters et al., 2004). Excitingly, the expression of VIMP increases following selenium supplementation in the liver of male mice, whereas in female mice VIMP expression only reaches a maximum after LPS challenge to induce an acute immune response (Stoedter et al., 2010). In regard to our data, this leads us to hypothesize that selenium supplementation and its potential sex-specific effects on VIMP expression might also result in a gender-biased effect on CD4 T cells.

Overall, using both hypothesis-free top-down computational analyses and bottom-up experimental methods, we have shown a regulatory role for the selenoprotein VIMP in controlling cytokine expression in CD4^+^CD25^-^ Teffs via several signaling pathways and transcriptional regulatory pathways. The same strategy should be generally extendable to other cell types in assisting the prediction and discovery of novel functions of any other genes of importance. In summary, our data identified an unrecognized critical regulatory role of the selenoprotein S (SELS/VIMP) in the inflammatory responses of human CD4^+^ Teffs. Our observation provides a viable insight into how dietary supplementation of selenium might mediate its effects on CD4^+^ Teffs and underscores the potential in therapeutically targeting VIMP in the treatment of various inflammatory and inflammation-related diseases.

### Limitations of the study

Although we have successfully demonstrated an unrecognized role for *VIMP* in the regulation of CD4 T cytokine expression and the underlying mechanisms, our study still presents certain limitations. As aforementioned, selenium supplementation and immune cell responses display a sexual dimorphism. In this study, we are aware that the majority of healthy donors were male. However, due to ethic regulations, we could not specify the gender of each individual donor, making it impossible to determine a possible sex-specific effect of *VIMP* on the effector functions of CD4 T cells.

In addition, our data is based on primary human CD4 T cells expanded *in vitro* and do not take into account all the complex cellular regulatory mechanisms directly and indirectly acting on CD4 T cells present *in vivo*. Our work has shown an intrinsic role of *VIMP* on human CD4 effector T cells, but to better elucidate the importance of our findings in a disease context, *Vimp*-deficient mice could have been used, which, however, were not available in our laboratory. Even though the whole-body deficiency of some selenoproteins is embryonically lethal (Santesmasses et al., 2020), *Vimp*-deficient mice have been recently reported and mainly used to study the role of *Vimp* in muscle functions (Addinsall et al., 2018, 2020; Wright et al., 2017). Excitingly, in line with our notion in human CD4 T cells, the reduction of *Vimp* expression even in heterozygous mice has been shown to increase the expression of several inflammatory genes in fast-twitch skeletal muscles (Wright et al., 2017).

### Resource availability

#### Lead contact

Further information and requests for different resources should be directed to and will be fulfilled by the lead contact, Feng He, Department of Infection and Immunity, Luxembourg Institute of Health, Esch-sur-Alzette, Luxembourg (feng.he@lih.lu).

#### Materials availability

The study did not generate any new unique materials.

#### Data and code availability

The whole-transcript microarray data have been deposited into Gene Expression Omnibus (GEO) repository with the access code https://www.ncbi.nlm.nih.gov/geo/query/acc.cgi?acc=GSE151266. Raw gel images from Figure 4C were deposited on Mendeley at https://doi.org/10.17632/6bd75yg6rp.1.

## Methods

All methods can be found in the accompanying Transparent methods supplemental file.

## Supplemental information

Document S1. Transparent methods and Figures S1–S3
