# Supplementary material for: Identification of VIMP as a gene inhibiting cytokine production in human CD4+ effector T cells
Source: iScience. 2021 Mar 9;24(4):102289. doi: 10.1016/j.isci.2021.102289 (PMC8024663; doi:10.1016/j.isci.2021.102289)
Supplement: Document S1. Transparent methods and Figures S1–S3 [file mmc1.pdf]

iScience, Volume *24*

Supplemental information

Identification of VIMP as a gene inhibiting cytokine production in human CD4+ effector T cells

Christophe M. Capelle, Ni Zeng, Egle Danileviciute, Sabrina Freitas Rodrigues, Markus Ollert, Rudi Balling, and Feng Q. He

Supplemental Information

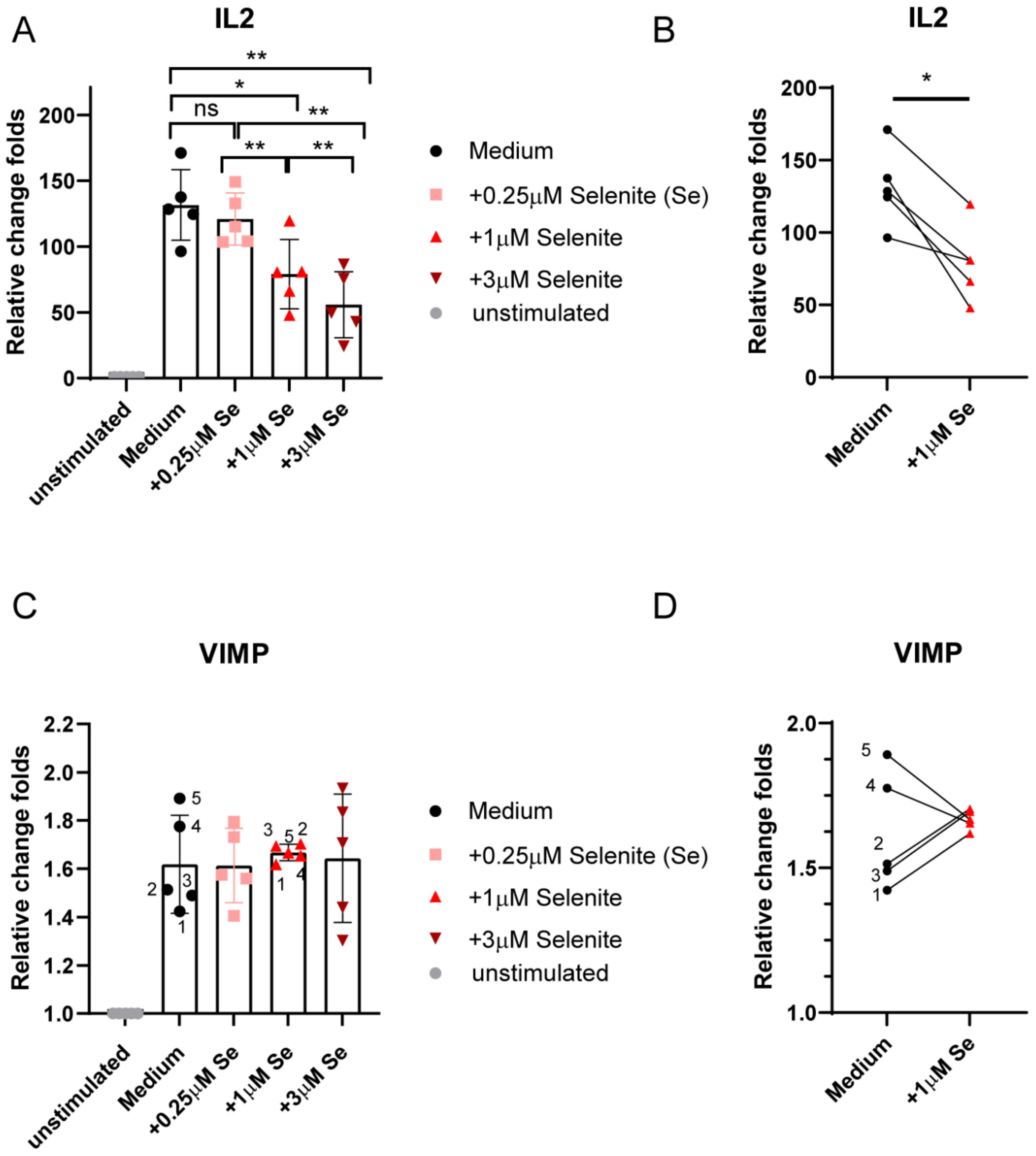

Supplemental Figure S1. Selenium supplementation suppresses IL2 production in CD4 Teffs, Related to Figure 2. Human CD4+CD25- T cells sorted from healthy donors were unstimulated in normal IMDM complete media or stimulated for 24 hrs by soluble anti-CD3/CD28 antibodies either in IMDM complete media alone or supplemented with different concentration of sodium selenite (S5261, Sigma Aldrich). (**A, C**) The mRNA expression of IL2 (**A**) and VIMP (**C**) was quantified by qPCR and normalized to the housekeeping gene RPS9 and to that of the unstimulated samples of the given donor. (**B, D)** A “zooming-in” analysis of the two selected concentrations of Se for IL2 (**B**) and VIMP (**D**). Each dot represents one heathy donor. The donor ID was indicated for the concentration of interests. Data are mean± standard deviation (s.d.). The P-values are determined

by a two-tailed paired Student’s t test. ns or unlabeled, non-significant; *P<=0.05, **P<=0.01 and ***P<=0.001.

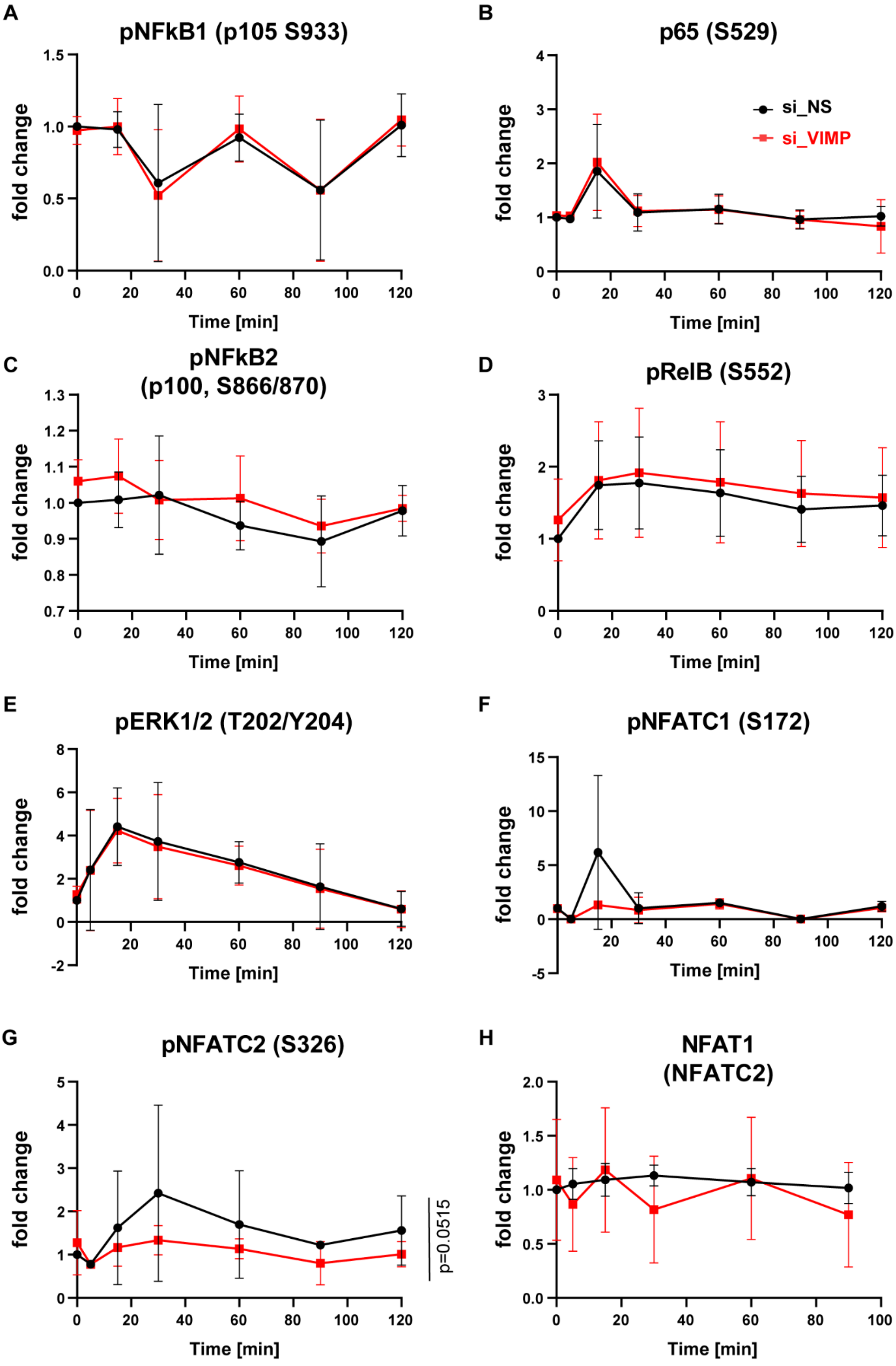
A
pNFkB1 (p105 S933)
fold change
Time [min]
B
p65 (S529)
si_NS
si_VIMP
fold change
Time [min]
C
pNFkB2
(p100, S866/870)
fold change
Time [min]
D
pRelB (S552)
fold change
Time [min]
E
pERK1/2 (T202/Y204)
fold change
Time [min]
F
pNFATC1 (S172)
fold change
Time [min]
G
pNFATC2 (S326)
fold change
Time [min]
p=0.0515
H
NFAT1
(NFATC2)
fold change
Time [min]

Supplemental Figure S2. VIMP knockdown only affects the phosphorylation of NFATC2, not the other major signaling pathways downstream of the TCR, Related to Figure 4.

Phosphorylation of proteins involved in the major signaling pathways downstream of TCR signaling in Teffs, assessed by flow cytometry at different time points following PMA/Ionomycin stimulation. Before stimulation, the cells were transfected with specific siRNA against VIMP (si_VIMP) versus non-specific siRNA (si_NS) for 1 day. (**G**) Only pNFATC2 was significantly decreased by VIMP knockdown. The other measured targets remain no significant change (**A**-**F**, **H**). The fold change was calculated by normalizing the geometric mean (Geomean) of the fluorescence intensities of all the conditions to that of the unstimulated control knockdown condition. Data are mean± standard deviation (s.d.). The P-values are determined by a two-tailed paired Student's t test over time including the data at different time points. ns or unlabeled, non-significant; *P<=0.05, **P<=0.01 and ***P<=0.001. All the graphs represent the pooled flow cytometry data for the fold change from 2-7 independent donors.

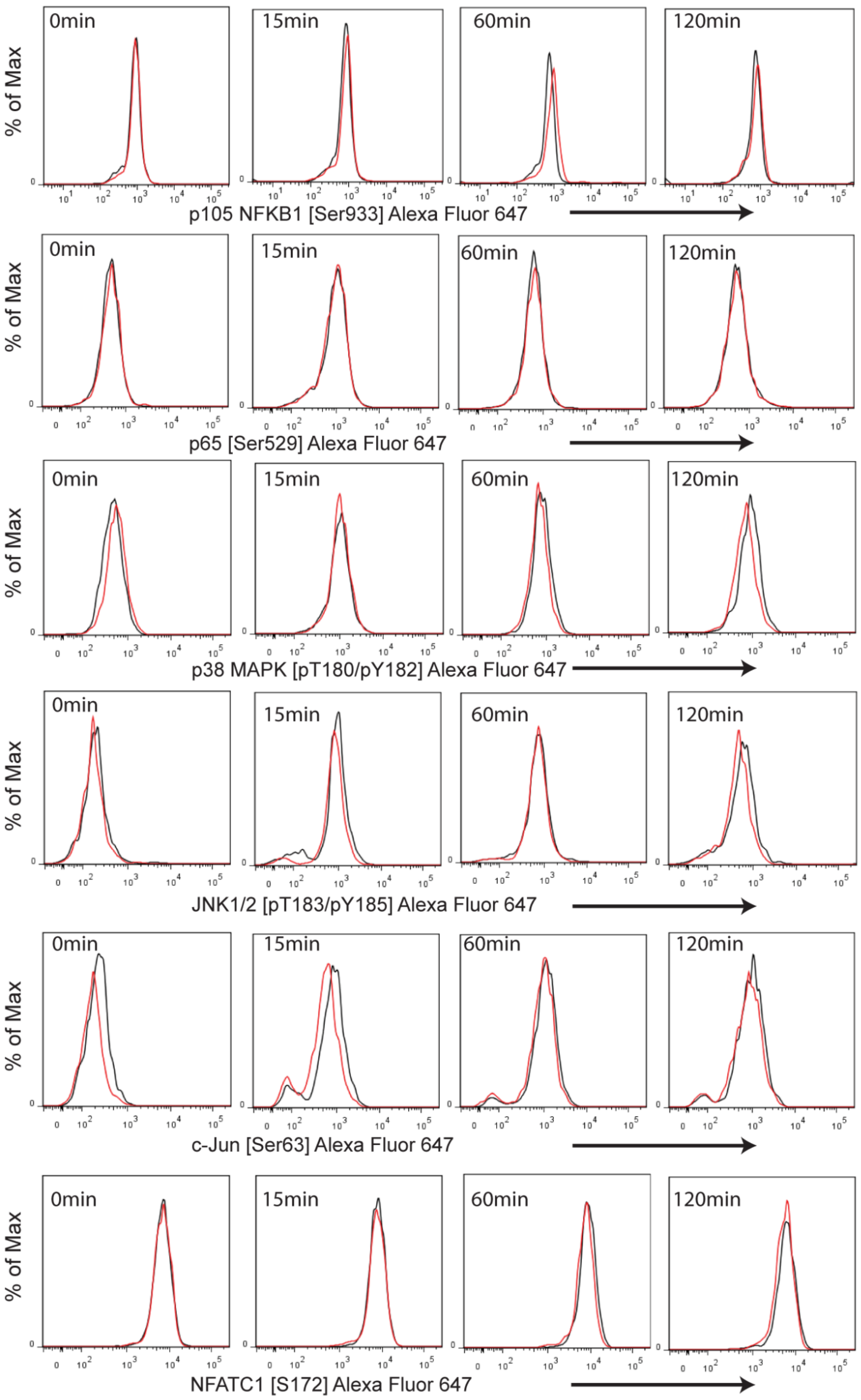

% of Max
0min
15min
60min
120min
p105 NFKB1 [Ser933] Alexa Fluor 647
p65 [Ser529] Alexa Fluor 647
p38 MAPK [pT180/pY182] Alexa Fluor 647
JNK1/2 [pT183/pY185] Alexa Fluor 647
c-Jun [Ser63] Alexa Fluor 647
NFATC1 [S172] Alexa Fluor 647

Supplemental Figure S3. VIMP knockdown does not affect other major signaling pathways downstream of the TCR, Related to Figure 4.

Representative histogram overlay for the phosphorylation of major signaling transduction proteins downstream of the TCR signaling in Teffs, assessed by flow cytometry at different time points following PMA/Ionomycin stimulation. Before stimulation, the cells were first transfected with specific siRNA against VIMP (si_VIMP) versus non-specific siRNA (si_NS) for 1 day. No significant effects on the phosphorylation levels of MAPK (p38, ERK1/2, cJun, JNK1/2) pathways and canonical (p65, p105) or non-canonical (RELB, NFκB2) NFκB pathways during the first 120 min stimulation after siRNA knockdown in Teffs. The expression of total NFAT1 protein was also unaffected by VIMP knockdown. The numbers in x-axis indicate the geometric mean (Geomean) fluorescence intensity of the different proteins or phosphorylation sites. Data are mean± standard deviation (s.d.). The other measured targets remain no significant change (**A**-**G**). The P-values are determined by a two-tailed paired Student's t test. ns or unlabeled, non-significant; *P<=0.05, **P<=0.01 and ***P<=0.001. All the graphs represent data from 2-7 independent donors.

Transparent Methods

Primary T cell isolation and culture

Buffy coats from healthy donors were provided by the Red Cross Luxembourg and the informed consent was obtained from each donor by the Red Cross Luxembourg. The T cell isolation and culture procedures have been described in our previous works (Danileviciute et al., 2019; He et al., 2012; Sawlekar et al., 2020). For the requirement of the STAR methods, we briefly described it here again. We added the RosetteSep™ Human CD4+ T cell Enrichment Cocktail (15062, Stemcell) to undiluted blood at a concentration of 50 µl/ml and incubated for 30 min at 4°C. The incubated samples were then diluted 2 times with FACS buffer (PBS + 2% FBS) and the CD4+ T cells were obtained following gradient centrifugation at 1200 g for 20 min, using Lympoprep (07801, StemCell) and SepMate™-50 tubes (85450, Stemcell). Before sorting, the CD4+ T cells were first stained with mouse monoclonal [RPA-T4] anti-human CD4 FITC (555346, BD Biosciences) (dilution 1:20), mouse monoclonal [M-A251] anti-human CD25 APC (555434, BD Biosciences) (dilution 1:20) and LIVE/DEAD® Fixable Near-IR Dead Cell Stain (L10119, Thermo Fisher Scientific) (dilution 1:500). Primary CD4 T cells ($CD4^{+}CD25^{-}$) were then sorted on a BD FACSAria™ III cell sorter (BD Biosciences).

Target	Fluorochromes	Dilution	Company	Clone	Reference
CD4	FITC	1:20	BD Biosciences	RPA-T4	555346
CD25	APC	1:20	BD Biosciences	M-A251	555434
Live/Dead	Near Infra-Red	1:500	Thermo Fisher Scientific	N.A.	L10119

Sorted human CD4+ T cells were cultured in IMDM (21980-032, Thermo Fisher Scientific) complete medium, supplemented with 10% heat-inactivated (56°C, 45 min) fetal bovine serum (FBS) (10500-064, Thermo Fisher Scientific), 1x Penicillin+Streptomycin (15070-063, Thermo Fisher Scientific), 1x MEM non-essential amino acids (M7145, Sigma-Aldrich) and 1x β-mercaptoethanol (21985-023,

Thermo Fisher Scientific). Every seven days for a maximum of four weeks, Teffs were derived from isolated $CD4^{+}CD25^{-}$ T cells by restimulating them with irradiated Epstein–Barr virus (EBV) transformed B-cells (EBV-B cells) (Probst-Kepper et al., 2009), at a 1:1 ratio to expand and maintain the culture. The EBV-B cells were irradiated in RS2000 X-Ray Biological Irradiator (Rad Source Technologies) for 30 min with a total of 90 Gy.

Teff siRNA knockdown and stimulation

Targeted gene's expression was knocked-down in up to 5 x 10^{6} cells using the P3 Primary Cell 4D-Nucleofector X Kit L (V4XP-3024, Lonza) with 90 µl P3 Primary cell solution and 100 pmol of corresponding si_RNA (resuspended in 10 ul RNAse-free H2O): si_Non-Specific scrambled control siRNA (si_NS or si_CTRL) (SC-37007, Santa Cruz), si_VIMP/SELS (SI03053512, Qiagen), si_E2F5 (SI00030436, Qiagen). siRNA transfection by electroporation was performed in the Amaxa 4D-Nucleofector™ X System (Lonza) according to the manufacturer's recommended program for primary human T cells (with the program code EO-115). Following transfection, the Teffs were first transferred into a 12-well plate with pre-warmed complete IMDM medium and incubated at 37 °C for 24 hrs before being stimulated with 25 µl/ml of soluble antibodies (Immunocult™ Human CD3/CD28 T Cell Activator) (10971, StemCell), or 10 ng/ml PMA (Phorbol 12-myristate 13-acetate, P8139, Sigma-Aldrich) and 100 ng/ml Ionomycin (I0634, Sigma-Aldrich) or Dynabeads® Human T-Activator CD3/CD28 for T Cell Expansion and Activation (11131D, Thermo Fischer Scientific) (with 1:1 ratio between number of cells and beads) in a 24-well plate for different specified time periods.

RNA extraction

The RNeasy Mini Kit (74106, Qiagen) was employed for RNA extraction following the manufacturer's instructions and including the digestion of genomic DNA with DNAse I (79254, Qiagen). The cells were lysed in RLT buffer (79216, Qiagen), supplemented with 1% beta-Mercaptoethanol (63689, Sigma-Aldrich). NanoDrop 2000c Spectrophotometer (Thermo Fisher Scientific) was used to measure RNA concentration. For the microarray analysis, the quality of RNA was first checked by assessing the RNA integrity number (RIN) using the Agilent RNA 6000 Nano kit (5067-1511, Agilent) and the Agilent 2100 Bioanalyzer Automated Analysis System (Agilent), according to the manufacture's protocol. Only the samples with RIN of 8.5 or higher were used in the further analysis.

Microarray measurement and analysis

The transcriptomic analysis of human effector T cells expanded from CD4+CD25- T cells isolated from the PBMCs of healthy donors were performed on the Affymetrix human gene 2.0 ST array at EMBL Genomics core facilities (Heidelberg, led by Dr. Benes Vladimir). The facility used 500 ng of total RNA in the protocol with the Ambion® WT Expression Kit (cat. 4411974) in order to obtain 10 ug of cRNA, which was then converted to ssDNA. 5.5 ug of ssDNA was labeled and fragmented using the WT Terminal Labeling, polyA and hyb Controls Kit (Affymetrix, cat. 901524). 3.75 ug of fragmented/labeled ssDNA (with hybridization controls) was hybridized to Affymetrix HuGene 2.0 Genechip at 45 °C for 16 hrs with rotation (60 rpm) and washed and stained on GeneChip Fluidics Stations 450 using GeneChip® Hybridization Wash and Stain Kit (Affymetrix, cat. 900720). Arrays were scanned using GeneChip Scanner 3000 7G with GeneChip Command Console software.

The expression signal at the exon level was summarized by the Affymetrix PLIER approach using the sketch approximation of quantile normalization with the option PM-GCBG (a GC content based background correction) using Affymetrix Expression Console v1.3.1.187. Before performing differential analysis, we first pre-processed the data with certain filtering steps. The filtering steps following the PLIER summary method included: 1) first removing any probeset whose cross-hyb type was not equal to 1; 2) removing any probeset corresponding to no identified gene or multiple genes according to the annotation (the file HuGene-2_0-st-v1.na33.2.hg19.transcript) and the library version r4 (May 23, 2012); 3), excluding the probesets with the average expression value in both groups (si_NS and si_VIMP) ≤ 2 times of the median value of the arrays (in our case, 2x the median was equal to the intensity value of 170); 4) if the mean intensity of the probesets in one group was higher, the number of absent calls among the three biological replicates should not be ≥ 1 in the group with higher mean intensity. To secure more robust analysis, we also analyzed the dataset using another model-based method (Berchtold et al., 2008; Weigand et al., 2012), i.e., RMA-sketch summary/normalization method (of note, the filtering steps mentioned above did not apply to the data resulted by the RMA-sketch summary method). We selected the probeset for further analysis only if the two-sided pair-wised T-test generated a P-value lower than 0.05 from the datasets summarized by both PLIER and RMA methods as demonstrated somewhere else (Weigand et al., 2012). To obtain a certain number of starting candidates, we lowered the threshold of the change fold to 1.2, which had to be recurrent in all the three donors, for our further analysis in consideration of both facts that VIMP is not a (co)transcription factor and the siRNA knockdown efficiency was not 100%. The database of mammalian transcription factors or cofactors, or chromatin remodeling factors was downloaded from the work of others (Zhang et al., 2012).

In this way, around 800 genes were significantly upregulated and around 550 genes were downregulated following VIMP knockdown, which were used for further analysis.

Correlation network and IPA

The Teff correlation network based on high-resolution time series datasets of Teffs was already calculated and constructed in our previous work (He et al., 2012) and we extracted the VIMP subnetwork for a deeper analysis in this work. Ingenuity Pathway Analysis (IPA) was used to reconstruct the regulatory network from the Ingenuity database following the instruction of provider (QIAGEN).

cDNA synthesis

The SuperScript™ IV First Strand Synthesis System (18091050, Thermo Fisher Scientific) was used for human cDNA synthesis using a maximum of 500 ng of RNA following the manufacturer's protocol. The master mix for the first step per sample including: 0.5 µl of 50 µM Oligo(dT)20 primers (18418020, Thermo Fisher Scientific), 0.5 µl of 0.09 U/µl Random Primers (48190011, Thermo Fisher Scientific), 1 µl of 10 mM dNTP mix (18427013, Thermo Fisher Scientific) and RNAse free water for a final volume of 13 µl in 0.2 ml PCR Tube Strips (732-0098, Eppendorf). The C1000 Touch Thermal Cycler (Bio-Rad) or UNO96 HPL Thermal Cycler (VWR) were employed with the following program: 5 min at 65 °C, followed by 2 min at 4 °C. For the second reaction step, the reaction mix was accompanied with 40 U RNaseOUT™ Recombinant Ribonuclease Inhibitor (10777019, Thermo Fisher Scientific), 200 U SuperScript™ IV Reverse Transcriptase (18090050, Thermo Fisher Scientific), a final concentration of 5mM Dithiothreitol (DTT) (707265ML, Thermo Fisher Scientific)

and 1x SuperScript™ IV buffer to reach a final reaction volume of 20 µl. We used the following thermocycler program for the second step: 10 min at 50 °C, then 10 min at 80 °C and at 4 °C until further usage. The nuclease-free water was used to dilute the obtained cDNA 5 times with a final volume of 100 µl.

Quantitative real-time PCR

The quantitative real-rime PCR (qPCR) reaction mix per sample enclosed: 5 µl of the LightCycler 480 SYBR Green I Master Mix (04707516001, Roche), 2.5 µl cDNA and 2.5 µl primers in a total reaction volume of 10 ul. The PCR reaction was performed in a LightCycler 480 (384) RT-PCR platform (Roche), using the LightCycler 480 Multiwell 384-well plates (04729749 001, Roche) sealed with the LC 480 Sealing Foil (04729757001, Roche). The program for qPCR used was as follows: 5 min at 95 °C; 45 cycles of (10 sec at 55 °C, 20 sec at 72 °C, 10 sec at 95 °C); melting curve (65-97 °C). The results were analyzed using the LightCycler 480 SW 1.5 software. Primers used for qPCR: RPS9 (QT00233989, Qiagen) as a reference gene, VIMP/SELS (QT00008169, Qiagen), IL2 (QT00015435, Qiagen), CSF2 (QT00000896, Qiagen), IL21 (QT00038612, Qiagen), CEBPG (QT00224357, Qiagen), E2F5 (QT00062965, Qiagen), IRX3 (QT00227934, Qiagen), RNF14 (QT00088291, Qiagen), ZBTB20 (QT00069776, Qiagen) and CTLA4 (QT01670550, Qiagen).

Western blotting

Novex™ WedgeWell 4-20% Tris-Glycine pre-casted gels (XP04202Box, Invitrogen) were used to run and separate proteins in the Novex™ Tris-Glycine SDS Running buffer (LC2675-4, Invitrogen). The proteins were then transferred (dry transfer) using an iBlot2™ Gel Transfer Device (IB21001, Invitrogen) and iBlot2™ PVDF stacks (IB24002, Invitrogen). Following the transfer, the membranes were blocked in 5% milk in PBS with 0.2% Tween20 (PBS-T) for 1 hr at room temperature with gentle shaking and incubated overnight at 4°C together with the primary antibodies, diluted in 5% BSA in PBS-T with 0.025% sodium azide. The next day, the membrane was washed three times (10 min each time) before and after incubation with secondary goat anti-rabbit HRP-coupled antibodies. The Amersham ECL Prime Western Blotting Detection Reagent (RPN2232, GE Healthcare Life Sciences) was used to detect the proteins and the image of the membranes was visualized on the ECL Chemocam Imager (INTAS). If necessary, the contrast and brightness of the obtained whole gel pictures was adjusted using *ImageJ*. The signal intensity of the protein bands was quantified using *ImageJ* and normalized to that of the housekeeping gene GAPDH. For the quantification of phospho proteins, both the phospho and the pan protein were normalized to GAPDH, before normalizing the phospho protein to the total protein.

**Target**	**Dilution**	**Company**	**Clone**	**Reference**
pNFATC2 (Ser326)	1:100	Sigma-Aldrich		SAB4503945
NFAT1	1:1000	Cell Signaling	D43B1	5861S
VIMP	1:1000	Sigma-Aldrich	Polyclone	V6639
GAPDH	1:200	Santa Cruz Biotechnology	FL-335	SC-25778
**Deposited gel data:**				
http://dx.doi.org/10.17632/6bd75yg6rp.1				

Proliferation assay

The proliferation of the Teffs was assessed using the CellTrace™ CFSE cell proliferation kit (C34554, Invitrogen). The final concentration of 1 µM CFSE dye was used in our work. To label the cells, they were incubated for exactly 2 min and 45 sec at RT in the dark. To stop the reaction, 10 ml FBS was added and the cells were centrifuged at 200 g for 10 min. After washing the cells in IMDM medium, the cells were subjected to the siRNA knockdown and counted. 10^5 Teff in a 96-well plate were used for each condition and stimulated for 2 days with a ratio of 1:1 of irradiated Epstein Barr Virus (EBV) B cells as previously described (He et al., 2012). After the stimulation, the cells were stained for living cells using LIVE/DEAD® Fixable Near-IR Dead Cell Stain (L10119, Thermo Fisher Scientific) (dilution 1:500) and acquired on a BD Fortessa™ analyzer. The data was analyzed in FlowJo 7.6.5.

Cytokine measurement by Mesoscale discovery (MSD) platform

The cell supernatant was collected after centrifugation of the cells (250 g, 10 min) and the selected list of secreted cytokines (CSF2, IL2, IL21) was measured in the undiluted cell culture medium using the MSD U-PLEX Human Biomarker group 1 kit (MSD, K15067L-1) and following the manufacturer's instructions. MESO QuickPlex SQ 120 instrument was used to read the plate and the data was analyzed with the MSD Workbench software.

Cytokine measurement by Cytometric Bead Array (CBA)

The cell supernatant was collected after centrifugation of the cells and the secreted IL2 in the diluted cell culture medium (1:4 dilution) was measured using the IL2 Flex set cytometric bead array (CBA) (BD, 558270) following the manufacturer's instructions. The acquisition was done on a BD Fortessa™ analyzer and the data was analyzed in FCAP Array™ v3.0.

PhosFlow cytometry analysis

Following stimulation, the cells were immediately fixed by adding the same volume of pre-warmed BD Cytofix Fixation Buffer (554655, BD) for 1 hr at 37 °C. After collecting the samples at all the different time points, they were then washed in FACS buffer and re-suspended in 200 µL of BD Phosflow Perm Buffer III (558050, BD) containing the antibodies for 30 min at 4 °C. After washing the cells with FACS buffer, they were re-suspended in FACS buffer to be acquired on the BD Fortessa™.

The antibodies used are the following (Table below): VIMP/SELS (V6639, Sigma-Aldrich) (dilution 1:200) with Goat Anti-rabbit IgG H&L Alexa Fluor® 647 (A-21245, Invitrogen) (dilution 1:200), NFAT1 FITC (611060, BD) (dilution 1:50), phospho p38 MAPK (T180/Y182) Alexa Fluor 647 (562066, BD) (dilution 1:50), Anti-Human phospho NFATC1 (pS172) mAb (MAB5640, R&D Systems) (dilution 1:400), phospho NFATC2 (NFAT1) (S326) (SAB4503945, Sigma-Aldrich) (dilution 1:800), PE-Cy7 Mouse anti-ERK1/2 (pT202/pY204) (560116, BD) (dilution 1:50), phospho JNK1/2 (T182/Y185) (dilution 1:200) (558268, BD), phospho cJun (S63) (9261S, Cell Signaling) (dilution 1:200), phospho p105 NFκB1 (S933) (4806S, Cell Signaling) (dilution 1:400), phospho p100 NFκB2 (S866/870) (4810S, Cell Signaling) (dilution 1:400), phospho p65 (S529) (558422, BD) (dilution 1:50), phospho RelB (S552) (4999S, Cell Signaling) (dilution 1:400) , Anti-Rabbit IgG H&L Alexa Fluor 647 (ab

150079, Abcam) (dilution 1:1000), APC Goat Anti-mouse IgG (minimal X-reactivity) (405308, Biolegend) (dilution 1:200). For the acquisition a BD Fortessa™ was used and the data was analyzed in FlowJo 7.6.5.

Target	Dilution	Company	Clone (if applicable)	Reference
Anti-VIMP/SELS	1:200	Sigma-Aldrich	Polyclone	V6639
FITC anti-NFATC2 (NFAT1)	1:50	BD Biosciences	1/NFAT-1	611960
Mouse anti-human pNFATC1 (pS172) MAb	1:400	R&D Systems	679340	MAB5640
APC Goat Anti-mouse IgG (minimal X-reactivity) Antibody	1:200	Biolegend	N.A.	405308
Alexa Fluor 647 Mouse anti-NFκB p65 (pS529)	1:50	BD Biosciences	K10-895.12.50	558422
PE-Cy7 Mouse anti-ERK1/2 (pT202/pY204)	1:50	BD Biosciences	20A	560116
Alexa Fluor 647 Mouse Anti-p38 MAPK (pT180/pY182)	1:50	BD Biosciences	36/p38	562066
phospho NFAT1/NFATC2 (S326)	1:800	Sigma-Aldrich	Polyclone	SAB4503945
phospho c-Jun (S63)	1:200	Cell Signaling	Polyclone	9261S
phospho JNK1/2 (T183/Y185)	1:200	BD Biosciences	Polyclone	558268
phospho p105 NFκB1 (S933)	1:400	Cell Signaling	18E6	4806S
phospho p100 NFκB2 (S866/870)	1:400	Cell Signaling	Polyclone	4810S
phospho RelB (S552)	1:400	Cell Signaling	Polyclone	4999S
Goat Anti-rabbit IgG H&L (Alexa Fluor® 647)	1:200	Invitrogen	N.A.	A-21245

Calcium/Ca2+ flux

To measure the calcium flux in Teffs, the cells were stained with mouse monoclonal [RPA-T4] anti-human CD4 FITC (555346, BD Biosciences) (dilution 1:100), LIVE/DEAD® Fixable Near-IR Dead Cell Stain (L10119, Thermo Fisher Scientific) (dilution 1:500) and the calcium dye Indo-1 (I1203, Thermo Fisher Scientific) (5 uM) for 60 min at 37 °C in complete IMDM medium as for the culture of Teffs. Following 3 washes with medium the cells were re-suspended in 300ul of medium and incubated for another 15-30 min at 37°C. The baseline of the calcium signal was measured for approximatively 30 sec before adding the soluble CD3/CD28 antibodies (1:40) (10971, StemCell) or 100 ng/ml Ionomycin (I0634, Sigma-Aldrich) to measure the activation-induced calcium flux. The cells were acquired on a BD Fortessa™ analyzer and the data was analyzed in FlowJo v10.5.

Ethics statement

The study procedures were approved by the ethic committee of the Red Cross Luxembourg. Informed consent was obtained from healthy blood donors through the Red Cross Luxembourg.

Statistical analysis

P values were calculated with paired two-tailed Student t test (Graphpad Prism or Excel) as specified in Figure legend. If the other test was used, it has also been specified in the corresponding Figure legend. All error bars represent the standard deviation.

Supplemental References

Berchtold, N.C., Cribbs, D.H., Coleman, P.D., Rogers, J., Head, E., Kim, R., Beach, T., Miller, C., Troncoso, J., Trojanowski, J.Q., *et al.* (2008). Gene expression changes in the course of normal brain aging are sexually dimorphic. Proc Natl Acad Sci U S A *105*, 15605-15610.

Danileviciute, E., Zeng, N., Capelle, C., Paczia, N., Gillespie, M.A., Kurniawan, H., Coowar, D., Vogt Weisenhorn, D.M., Giro, G.G., Grusdat, M., *et al.* (2019). PARK7/DJ-1 promotes pyruvate dehydrogenase activity and maintains Treg homeostasis. https://doi.org/10.1101/2019.1112.1120.884809.

He, F., Chen, H., Probst-Kepper, M., Geffers, R., Eifes, S., Del Sol, A., Schughart, K., Zeng, A.P., and Balling, R. (2012). PLAU inferred from a correlation network is critical for suppressor function of regulatory T cells. Molecular systems biology *8*, 624.

Probst-Kepper, M., Geffers, R., Kroger, A., Viegas, N., Erck, C., Hecht, H.J., Lunsdorf, H., Roubin, R., Moharregh-Khiabani, D., Wagner, K., *et al.* (2009). GARP: a key receptor controlling FOXP3 in human regulatory T cells. J Cell Mol Med *13*, 3343-3357.

Sawlekar, R., Magni, S., Capelle, C., Baron, A., Zeng, N., Mombaerts, L., Yue, Z., Yuan, Y., He, F.Q., and Gonçalves, J. (2020). Causal dynamical modelling predicts novel regulatory genes of FOXP3 in human regulatory T cells. bioRxiv, 2020.2002.2013.943688.

Weigand, J.E., Boeckel, J.N., Gellert, P., and Dimmeler, S. (2012). Hypoxia-induced alternative splicing in endothelial cells. PLoS One *7*, e42697.

Zhang, H.M., Chen, H., Liu, W., Liu, H., Gong, J., Wang, H., and Guo, A.Y. (2012). AnimalTFDB: a comprehensive animal transcription factor database. Nucleic Acids Res *40*, D144-149.
